# Hemodynamic Correlates of Electrophysiological Activity in the Default Mode Network

**DOI:** 10.3389/fnins.2019.01060

**Published:** 2019-10-04

**Authors:** Marco Marino, Giorgio Arcara, Camillo Porcaro, Dante Mantini

**Affiliations:** ^1^Brain Imaging and Neural Dynamics Research Group, IRCCS San Camillo Hospital, Venice, Italy; ^2^Institute of Cognitive Sciences and Technologies (ISTC) – National Research Council (CNR), Rome, Italy; ^3^S. Anna Institute and Research in Advanced Neurorehabilitation (RAN), Crotone, Italy; ^4^Department of Information Engineering, Università Politecnica delle Marche, Ancona, Italy; ^5^Research Center for Motor Control and Neuroplasticity, KU Leuven, Leuven, Belgium

**Keywords:** resting state, high-density EEG, fMRI, DMN, alpha rhythm

## Abstract

Hemodynamic fluctuations in the default mode network (DMN), observed through functional magnetic resonance imaging (fMRI), have been linked to electrophysiological oscillations detected by electroencephalography (EEG). It has been reported that, among the electrophysiological oscillations, those in the alpha frequency range (8–13 Hz) are the most dominant during resting state. We hypothesized that DMN spatial configuration closely depends on the specific neuronal oscillations considered, and that alpha oscillations would mainly correlate with increased blood oxygen-level dependent (BOLD) signal in the DMN. To test this hypothesis, we used high-density EEG (hdEEG) data simultaneously collected with fMRI scanning in 20 healthy volunteers at rest. We first detected the DMN from source reconstructed hdEEG data for multiple frequency bands, and we then mapped the correlation between temporal profile of hdEEG-derived DMN activity and fMRI–BOLD signals on a voxel-by-voxel basis. In line with our hypothesis, we found that the correlation map associated with alpha oscillations, more than with any other frequency bands, displayed a larger overlap with DMN regions. Overall, our study provided further evidence for a primary role of alpha oscillations in supporting DMN functioning. We suggest that simultaneous EEG–fMRI may represent a powerful tool to investigate the neurophysiological basis of human brain networks.

## Introduction

The default mode network (DMN) is a large-scale brain network comprising a specific constellation of cortical regions, including left and right angular gyrus (LANG and RANG), posterior cingulate cortex (PCC), and medial prefrontal cortex (MPFC). The activity of DMN typically decreases during performance of goal-directed cognitive tasks and increases during resting state (Raichle et al., 2001), undertaking a relevant role in mediating the communication with other brain networks (Mantini et al., 2013), and in modulating attention processes from external to internal sources (Goldman et al., 2002; Laufs et al., 2003a; Hanslmayr et al., 2011; Mo et al., 2013).

DMN functioning has been investigated using slow hemodynamic fluctuations observed through functional magnetic resonance imaging (fMRI) (Greicius et al., 2003). fMRI has proved to be very accurate in displaying brain activations with high spatial resolution, of a few millimeters (Ogawa et al., 1990; Biswal et al., 1995), but inadequate for directly detecting the fast oscillations associated with neuronal processes. This limitation has boosted the interest toward the simultaneous combination of fMRI with electroencephalography (EEG), which, contrary to fMRI, provides a direct measure of electrophysiological activity, with high temporal resolution, on the scale of milliseconds (Nunez and Silberstein, 2000). Starting from the evidence that EEG and fMRI measurements reflect the same brain activity (Logothetis et al., 2001; Eichele et al., 2009; Jorge et al., 2014), simultaneous EEG–fMRI was used to shed light on the hemodynamic correlates of electrophysiological DMN activity (Laufs et al., 2003a; Mantini et al., 2007; Scheeringa et al., 2012; Mayhew et al., 2013a; Mo et al., 2013).

To explore the hemodynamic correlates of electrophysiological activity, EEG neural power is typically calculated in one or more frequency bands, convolved with a canonical hemodynamic response function (HRF), and correlated with fMRI signals (Goldman et al., 2002; Laufs, 2008). Notably, this approach has been extensively used for investigating brain activity especially in relation to alpha, which is known to be the dominant idling electroencephalographic rhythm during resting state. In most of these studies, alpha power oscillations were calculated from occipital EEG sensors and correlated with fMRI time series from all voxels in the brain (Goldman et al., 2002; Laufs et al., 2003a; Scheeringa et al., 2012; Mo et al., 2013). Widespread negative alpha power correlation was mainly found in brain regions involved in attention processes (Laufs et al., 2003a), and belonging to the visual system (Goldman et al., 2002; Scheeringa et al., 2012; Mayhew et al., 2016), whereas positive alpha power correlation was reported in brain regions belonging to the DMN (Jann et al., 2009; Knyazev et al., 2011; Mo et al., 2013). In a previous study (Mantini et al., 2007), we further explored the electrophysiological correlates of resting state brain networks, by considering all the other frequency bands, including delta, theta, beta, and gamma. We found that the DMN presents a specific electrophysiological signature, which involves the coalescence of different electrical oscillations, among which alpha and beta rhythms play a dominant role (Mantini et al., 2007). Other studies reported significant correlations of DMN activity with other frequency bands, including positive beta power correlation in PCC and dorsal MPFC (Laufs et al., 2003b), and negative theta power correlation in MPFC (Scheeringa et al., 2008). All these results, however, have the common drawback of being derived from EEG power oscillations evaluated at the sensor level. In fact, approaches based on EEG recordings do not enable an exact match between EEG and fMRI measurements in a common reference system, i.e., the brain volume (Laufs, 2008; Ostwald et al., 2011). Recent technological advances enabled the detection of the DMN using source reconstructed high-density EEG (hdEEG) data (Liu et al., 2017, 2018; Marino et al., 2019). These opened the way for the investigation of band-limited neural power at the source level (Samogin et al., 2019). Notably, source-space analyses of hdEEG data in simultaneous EEG–fMRI studies could enable the direct comparison between electrophysiological and hemodynamic activity. This may enable the characterization of the brain rhythms contributing to hemodynamic activity in the DMN, as well as other brain networks.

In this study, we aimed to test the hypothesis that different brain rhythms contribute to DMN functioning. In particular, we postulate that DMN spatial configuration closely relates to the specific neuronal oscillations considered, and that alpha rhythm, more than other frequency bands, correlates with hemodynamic activity in brain regions belonging to the DMN nodes.

## Materials and Methods

### Subjects and Experimental Design

Eyes-open resting EEG and fMRI data were simultaneously acquired for 10 min in 20 healthy young adults volunteers (age 24 ± 3.3 years, 10 females). All participants reported normal or corrected-to-normal vision, and had no psychiatric or neurological history. Before undergoing the examination, they gave their written informed consent to the experimental procedures, which were approved by the Medical Ethics Committee of the UZ Leuven.

### EEG Data Acquisition

Electroencephalography signals were recorded by the MR-compatible 256-channel HydroCel Geodesic Sensor Net (GSN) (EGI, Eugene, OR, United States). The impedance of each electrode was maintained lower than 50 kΩ across the full recording, in line with recommendations for the HydroCel GSN, by soaking the sponge contained in each electrode with a saline solution. In order to maintain the contact of the EEG electrodes with the patient scalp, an elastic bandage was placed above the EEG net. The electrocardiographic (ECG) signal was also acquired by using two MR-compatible electrodes positioned on the chest, in correspondence to the apical and the left side of the heart, respectively. The EEG and ECG cables were connected to the EEG amplifier, which was contained in a field isolation containment system (FICS) and positioned next to the MR bore. EEG data were recorded at a sampling rate of 1 KHz, and were synchronized to the MR scanner internal clock. The collected signals were sent via an optical cable to the EEG recording computer outside the MR scanner room.

### MRI Data Acquisition

Functional magnetic resonance imaging data acquisition was performed using a 3T Philips Achieva MR scanner (Philips Medical Systems, Best, Netherlands) using a T2^∗^-weighted SENSE sequence. The scanning parameters were TR = 2000 ms, TE = 30 ms, 36 slices, 80 × 80 matrix, voxel size 2.75 × 2.75 × 3.75 mm^3^, flip angle = 90°. During simultaneous EEG–fMRI recordings, the helium pump of the magnet was switched off for the full duration of the functional acquisition. A T1-weighted whole-head structural MR image (sMRI), to be used for head tissue modeling, was collected with a turbo field echo sequence with the following scanning parameters: TR = 8.25 ms, TE = 3.8 ms, flip angle = 8°, voxel size: 1 mm^3^ isotropic. A T1-weighted whole-head ultrashort echo time (UTE) image, to be used for electrode localization, was collected with a fast field echo sequence with the following scanning parameters: TR = 8 ms, TE = 0.14 ms, flip angle = 10°, voxel size: 1 mm^3^ isotropic.

### MRI Data Processing

Processing of MRI data was carried out using built-in MATLAB (MathWorks, Natick, MA, United States) functions and the SPM12 software^1^. sMRI data preprocessing included intensity non-uniformity (INU) correction and image segmentation, which were carried out by using the unified segmentation algorithm implemented in SPM12, using a regularization parameter equal to 0.0001 and a smoothing parameter equal to 40 mm FWHM. UTE images were processed using a new method for EEG electrodes localization from MR images (Marino et al., 2016). This procedure consists of an image-processing step to improve image quality, perform image segmentation and detect the head shape, and an electrode-detection and -labeling step. In the first part, a search volume is defined around the external border of the head, i.e., the scalp, where the electrodes are positioned. Secondly, candidate electrodes are identified within the search volume in the UTE images, filtered, and matched with template EEG points, allowing for direct electrode labeling. fMRI data were preprocessed by means of an automated pipeline developed using SPM12, including motion correction, spatial alignment to sMRI, bias field correction, co-registration to standard space, and spatial smoothing at 6 mm full width half maximum (Mantini et al., 2013). The fMRI images were analyzed to obtain DMN spatial maps from each individual, as well as a DMN group-level map. Connectivity analysis was performed, separately for each subject, using spatial independent component analysis (sICA), which was used for decomposing the fMRI data into brain activity patterns starting from the spatial covariance of the measured signals (McKeown et al., 1998). We estimated the number of ICs by using the minimum description length criterion (Calhoun et al., 2001). Accordingly, 26–43 ICs were extracted, depending on the specific fMRI dataset. ICs were calculated using the FastICA algorithm, with a deflation approach and hyperbolic tangent non-linearity (Esposito et al., 2005). For each IC, a spatial map and an associated time series are extracted. The spatial map expresses the intensity of the activity across the voxels of that pattern, whereas the time series corresponds to its course over time (Mantini et al., 2007, 2009). The spatial map was converted to *z*-scores by subtracting the average intensity across voxels, and dividing the resulting map by the standard deviation across voxels. The IC corresponding to DMN was identified using an automated template-matching procedure, in which the considered DMN-template was derived from our previous fMRI study (Mantini et al., 2013). Specifically, the DMN was identified as the IC showing the highest spatial correlation with the DMN template map in Montreal Neurological Institute (MNI) space. We derived DMN group-level correlation map by performing a one-sample one-sided *t*-test, using a mass-univariate analysis on the individual DMN maps. According to this approach, each voxel displayed as significant in the results indicates that there was significant correlation at the group level. We corrected the significance level for multiple comparisons (for multiple voxels involved in the analysis) between single-subject z-scores correlation maps using the Benjamini-Yekutieli false discovery rate (BY-FDR) procedure (Benjamini and Yekutieli, 2001), which does not make any assumptions about sample dependency. The significance threshold for the DMN group-level correlation map derived from the fMRI data was set to *p* < 0.05, BY-FDR corrected.

### EEG Data Processing

**FIGURE 1:**
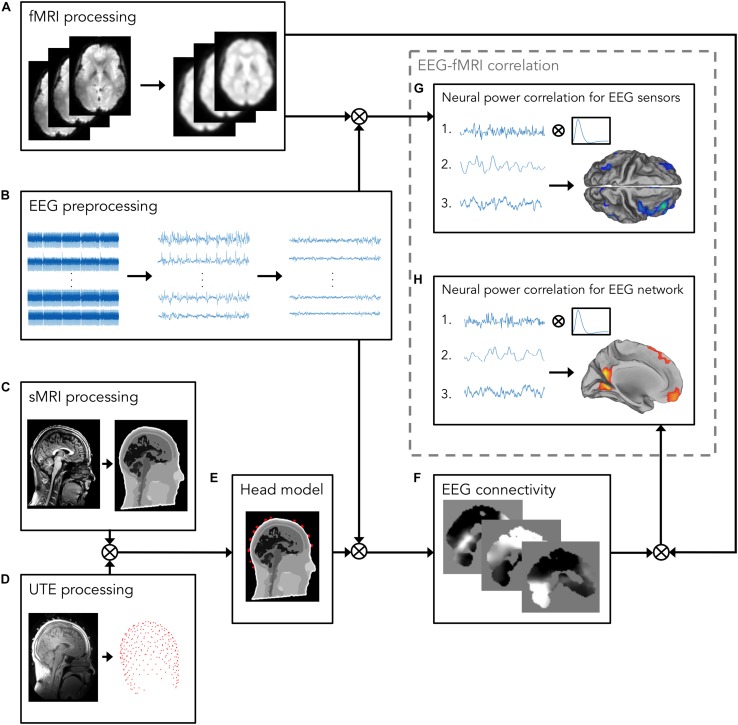
Analysis workflow for investigating the relationship between EEG and fMRI measurements. The workflow consists of the following processing steps: **(A)** fMRI processing, including motion correction, spatial alignment to sMRI, bias field correction, co-registration to standard space, and spatial smoothing. The resulting fMRI signals will be used for the EEG–fMRI correlation analysis; **(B)** EEG preprocessing, including removal of MR-related artifacts, i.e., gradient artifact, displayed on the left, and BCG, displayed in the middle, and removal of other biological artifacts, e.g., EOG, and EMG. On the right, artifacts-free EEG recordings are shown; **(C)** sMRI processing, including INU correction and image segmentation in 12 head tissues; **(D)** UTE processing for detecting EEG electrode positions; **(E)** Head model, generated by integrating the information about EEG electrode positions and segmented sMR image, in which electrical properties are assigned to each head tissue. Following source reconstruction, in which cleaned EEG data are combined with the realistic head model, **(F)** EEG connectivity analysis is performed. To this end, the power in alpha band is calculated from source-reconstructed EEG signals. Then, power signals are given in input to an ICA algorithm for separating patterns, each consisting of a spatial map and a time-course, of coordinated activity in the brain and artifactual sources. From these patterns, the one associated with the DMN is automatically selected using a template-matching procedure; **(G,H)** EEG–fMRI correlation analysis; **(G)** The power in alpha band from occipital channels (1) is calculated from artifact-free EEG recordings obtained in panel **(B)**, and, after convolving it with a canonical HRF (2), it is correlated with the fMRI signals processed in panel **(A)** (3); **(H)** the DMN time-course (1), calculated in panel **(F)**, is convolved with a canonical HRF (2), and correlated with the fMRI signals processed in panel **(A)** (3).

EEG data were processed using built-in MATLAB (MathWorks, Natick, MA, United States) functions and the EEGLAB toolbox^2^ (Delorme and Makeig, 2004) (Figure 1). EEG signals acquired during simultaneous fMRI scanning are affected by various artifacts (Yan et al., 2009; Marino et al., 2018a), which contaminate the EEG signal changes associated with neuronal activity. The EEG data processing pipeline proposed in this work (Figure 1) enabled the removal of MR-related artifacts and optimized the generation of the realistic head model, by using advanced head modeling techniques, including accurate information about EEG electrode positions.

We first attenuated MR-related artifacts using the fMRI artifact template removal (FASTR) method implemented in EEGLAB (Niazy et al., 2005), and the ballistocardiographic (BCG) artifact by means of the adaptive optimal basis set (aOBS) method (Marino et al., 2018b). Then, hdEEG data were given as input to an automated analysis workflow we previously developed and validated for brain network reconstruction from hdEEG data (Liu et al., 2017, 2018; Marino et al., 2019). This in-house processing pipeline consists of the following steps: (1) signal preprocessing, (2) head model generation, (3) source reconstruction, and (4) connectivity analysis. As for signal preprocessing, channels with poor signal quality were first identified based on either the Pearson correlation, in the frequency band (1–80 Hz), between each channel signal and the signal from all the other channels, or according to the variance of each channel’s noise, estimated in the frequency band (200–250 Hz) where the contribution of the brain activity can be considered negligible. Bad channels were defined as those channels for which at least one of the two abovementioned parameters was an outlier as compared to its total values distribution. These bad channels were corrected by interpolating the time courses from the neighboring channels. Then, EEG data were filtered in the frequency band (1–80 Hz), and ICA was applied to remove biological artifacts, including electrooculographic (EOG) and electromyographic (EMG) artifacts, from the EEG recordings. Independent components (ICs) were estimated with a fast fixed-point ICA (FastICA) algorithm based on a deflation approach and hyperbolic tangent as contrast function (Mantini et al., 2008). Artifactual ICs were automatically classified based on three parameters, including correlation values between ICs and reference EOG and EMG signals, similarity of ICs power spectrum with a 1/*f* function, and kurtosis of ICs timecourse. Following artifacts rejection, EEG signals were re-referenced in average reference (Liu et al., 2015). For head model generation, the integration of information about subject’s head geometry, tissues electrical properties, and EEG electrodes position is required. Head geometry was derived from the participant’s sMRI. A high-resolution head template in MNI space, segmented into 12 tissue classes (skin, eyes, muscle, fat, spongy bone, compact bone, cortical gray matter, cerebellar gray matter, cortical white matter, cerebellar white matter, cerebrospinal fluid, and brain stem) (Haueisen et al., 1997) was warped to subject space, with a non-linear deformation calculated using the normalization tool in SPM12 (as in Liu et al., 2017). The conductivity of the different head tissues was defined based on previous literature (Liu et al., 2017, 2018; Samogin et al., 2019). EEG electrode positions, extracted from the participant’s UTE image (Marino et al., 2016), were rigidly co-registered to the individual head shape. Following EEG electrodes position co-registration and head tissues segmentation, the leadfield matrix, which translates the activation of each assumed brain source to scalp electrical potentials, was calculated by using SimBio^3^ (Wolters et al., 2004). For source reconstruction, preprocessed hdEEG signals and generated head model were given as input to the exact low-resolution brain electromagnetic tomography (eLORETA) algorithm (Pascual-Marqui et al., 2011), to estimate brain activity in the source space, defined by a 6 mm grid spanning the whole cortex. Since brain activity in the source space is expressed, for each voxel, with three dimensions, power time-courses were computed by summing up, at each time point, the power calculated along each direction. Following hdEEG signals source reconstruction, for each time-course in the gray matter, we calculated the short-time Fourier transform using a Hamming window of 2 s, with 50% overlap between consecutive windows, to reconstruct power in delta (1–4 Hz), theta (4–8 Hz), alpha (8–13 Hz), beta (13–30 Hz), and gamma (30–80 Hz), and full (1–80 Hz) band, at steps of 1 Hz. Then, we performed connectivity analysis, separately for each subject, using temporal ICA (tICA) (Marques et al., 2009; Formaggio et al., 2011; Yuan et al., 2016). The number of ICs was estimated by using the minimum description length criterion (Calhoun et al., 2001). Accordingly, 19 to 44 ICs were extracted, depending on the specific hdEEG dataset. ICs were calculated using the FastICA algorithm, with a deflation approach and hyperbolic tangent non-linearity (Esposito et al., 2005). Following tICA decomposition, we obtained a set of temporally ICs. The IC time-courses were temporally correlated with the band-limited power in each brain voxel, thereby obtaining a spatial map associated with the IC (Brookes et al., 2011). *Z*-score maps at the single-subject level were derived by applying the Fisher’s *r*-to-*z* transform to the correlation maps. The IC corresponding to DMN was identified using an automated template-matching procedure, in which the considered DMN template was derived from previous hdEEG studies (Liu et al., 2017, 2018). Specifically, the DMN-template was warped to the individual MR space, and the DMN was identified as the IC showing the highest spatial correlation with the DMN template map in individual space. The whole procedure for EEG data processing was run separately for each subject.

### Neural Power Correlation for EEG Sensors

In order to replicate findings from previous studies, we extracted the alpha power from channels located in the occipital cortex (Goldman et al., 2002; Laufs et al., 2003a; Scheeringa et al., 2012). Alpha power was calculated from EEG signals, preprocessed using the automated pipeline described above. Then, to assess the relationship between electrophysiological and hemodynamic measurements, these power time-courses were convolved with a canonical HRF (Friston et al., 1998), and correlated voxel-by-voxel with the fMRI signals simultaneously acquired with the EEG data. The resulting correlation map was transformed to z-scores using the Fischer’s *r*-to-*z* transform, as previously done for the DMN map. In particular, for each subject, the average of the EEG signals was correlated with the fMRI time-course for each voxel. fMRI data were previously upsampled to the EEG power temporal resolution, i.e., one sample per second. We derived group-level correlation maps by stacking the z-score maps of each subject. To assess the statistical significance of the z-score maps at the group-level, we performed a one-sample one-sided *t*-test, separately for each voxel, according to a mass-univariate approach. Significance for each voxel was defined as the result of the one-sided *t*-test considering 20 observations, i.e., the number of participants, and the z-scores as dependent variable. We conducted separate mass-univariate analyses for each frequency band, and we corrected the significance level for multiple comparisons (due to the involvement of multiple voxels and different frequency bands) using the BY-FDR procedure (Benjamini and Yekutieli, 2001), as also done for the fMRI-derived DMN. The significance threshold was set to *p* < 0.05, BY-FDR corrected.

### Neural Power Correlation for EEG Networks

To study the relationship between networks identified from EEG data and fMRI signals, we focused on the electrophysiological characterization of hemodynamic DMN. Thus, for each frequency band, we used the power time-course associated with DMN time-course obtained from source-reconstructed EEG data. This was convolved with a canonical HRF and correlated voxel-by-voxel with the fMRI signals (Yuan et al., 2016). fMRI data were previously upsampled to the EEG power temporal resolution, i.e., one sample per second. A group-level analysis was carried out using the same statistical approach described in the previous section. The significance threshold for correlation maps between EEG network neural power and voxel-by-voxel fMRI signals was set to *p* < 0.05, and BY-FDR corrected. To assess the spectral specificity of the hemodynamic DMN, we quantified the spatial correspondence between the spatial map of the fMRI-derived DMN and the spatial pattern obtained correlating the hdEEG-derived DMN time-course with the fMRI signals. To this end, we used the Pearson correlation, i.e., correlation coefficient (CC), and the dice coefficient (DC). The latter is an index that ranges between 0 and 1, and is equal to 0 when there is no overlap between patterns and is instead equal to 1 when the patterns are perfectly overlapping.

## Results

To disentangle the relationship between EEG and fMRI measurements during resting state, we used hdEEG data simultaneously acquired during fMRI scanning. We first looked at the correlation between EEG neural power calculated from occipital sensors and fMRI signals to replicate findings from previous studies. Then, we investigated the link between EEG neural power associated with hdEEG-derived DMN and fMRI signals to identify the hemodynamic correlates of electrophysiological activity in the DMN.

**FIGURE 2:**
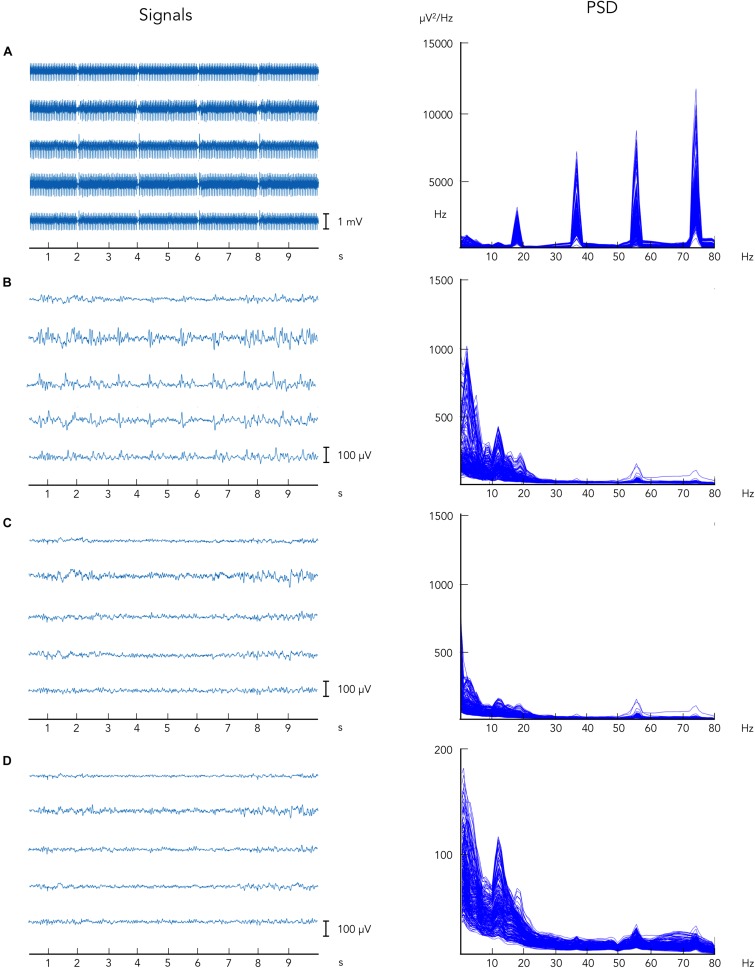
Illustrative example of EEG data collected during simultaneous EEG–fMRI following the artifacts removal steps. EEG data are displayed: **(A)** before gradient artifact correction, i.e., raw data, **(B)** after gradient artifact correction, **(C)** after BCG artifact correction, and **(D)** after ICA-based artifacts removal. On the left, EEG signals are reported for five representative channels. On the right, power spectrum density is shown for all EEG recording channels.

Ballistocardiographic artifact was successfully reduced following the application of the aOBS method, as shown in Figure 2, left. Applying ICA after the attenuation of MR-related artifacts, when non-stationary artifactual sources were not present anymore, enabled the removal of other biological artifacts, including EOG and EMG, and gradient artifact residuals. Following the application of each preprocessing step, we reported progressive artifacts removal and consistent neuronal signal preservation as also revealed by the profile of power spectra, in which only a slightly reduction in the peak in alpha power was visible (Figure 2, right).

### Neural Power Correlation for EEG Sensors

**FIGURE 3:**
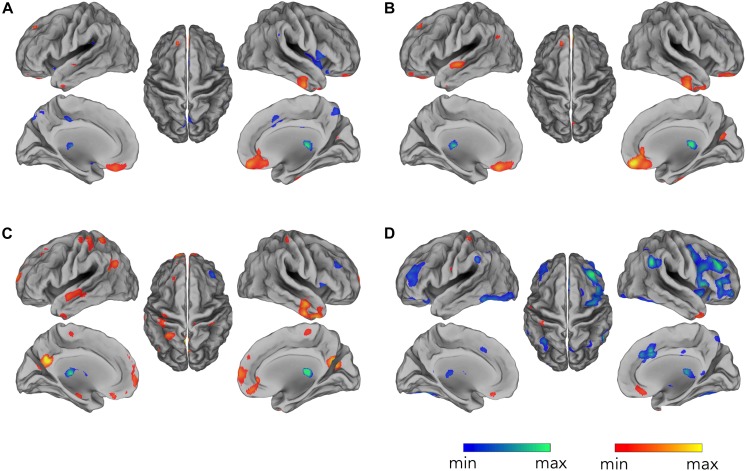
Occipital EEG alpha power correlating with fMRI signals. Correlation maps are displayed for EEG data: **(A)** before gradient artifact correction, i.e., raw data, **(B)** after gradient artifact correction, **(C)** after BCG artifact correction, and **(D)** after ICA-based artifacts removal. Group-level correlation maps between occipital alpha power time-course and voxel-by-voxel fMRI signals (*n* = 20, significance level *p* < 0.05 BY-FDR corrected).

By correlating EEG occipital sensors power with voxel-by-voxel fMRI signals, for the alpha band, we found widespread negative correlation in both left and right frontal and parietal lobes, displaying spatial patterns resembling the ones typically characterizing attention-related networks (Figure 3D) (*p* < 0.05, BY-FDR corrected), and in the left and right low-lateral occipital cortex (Figure 3D), in areas belonging to the visual system. These correlation patterns were clearly depicted only following the complete application of all the artifact removal steps, whereas we did not report any significant negative correlations at the group level for the intermediate steps (Figures 3A–C). On the other hand, we found positive correlation roughly in the ventral-MPFC, which emerged following the correction of the gradient artifact and it was consistently present following each subsequent artifact removal step.

### Neural Power Correlation for EEG Networks

**FIGURE 4:**
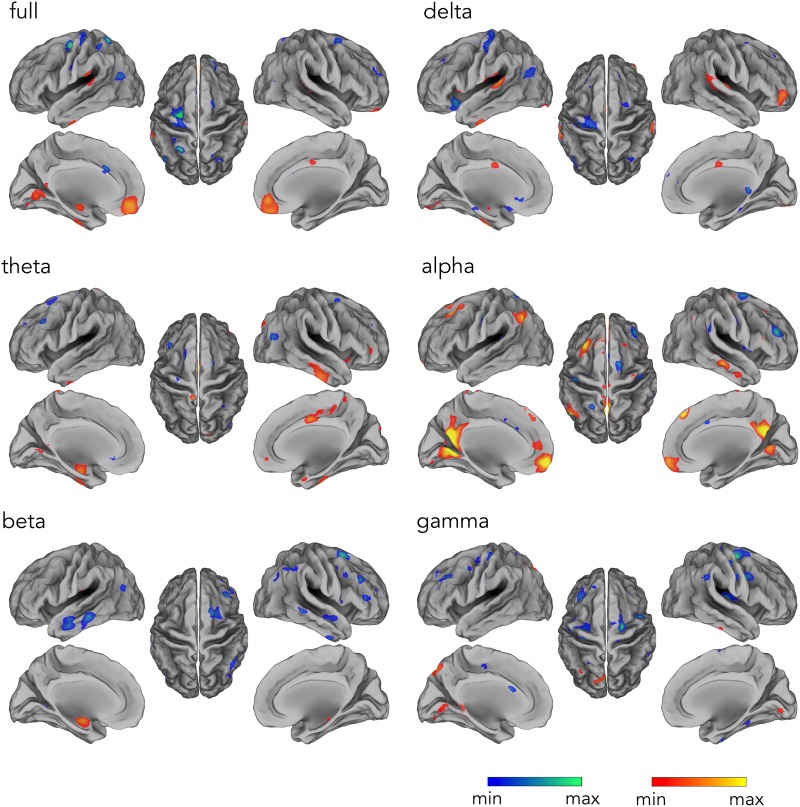
Temporal profile of the hdEEG-derived DMN activity correlating with fMRI signals in delta (1–4 Hz), theta (4–8 Hz), alpha (8–13 Hz), beta (13–30 Hz), and gamma (30–80 Hz), and full (1–80 Hz) band. Group-level correlation maps between DMN time-course and voxel-by-voxel fMRI signals (*n* = 20, significance level *p* < 0.05 BY-FDR corrected).

Our analysis workflow for source reconstructing hdEEG data enabled the detection of the DMN for EEG recordings simultaneously acquired with fMRI scanning, as previously achieved with hdEEG data acquired outside the MR environment (Liu et al., 2017). Next, we analyzed the hemodynamic correlates of electrophysiological DMN activity in different frequency bands. The time-course corresponding to DMN IC was correlated with the fMRI signal waveforms on a voxel-by-voxel basis. By correlating voxel-by-voxel fMRI signals with the time-course associated with hdEEG-derived DMN spatial map, we found widespread significant positive correlation for alpha and full band (Figure 4) (*p* < 0.05, BY-FDR corrected). In particular, the correlation patterns displayed a spatial distribution specifically depicting DMN areas, including PCC and MPFC (for full band), and also LANG and RANG (only for alpha band) (Figure 4). Also, negative correlations were present in ventral anterior cingulate cortex (ACC) for alpha, beta, and for the full band, but not for the other frequency bands. Furthermore, we noticed that the spatial configuration of the hdEEG-derived DMN largely depended on the frequency band considered, with a foremost contribution given by the alpha band, as shown by the highest CC and DC values, 0.64 and 0.58, respectively (Table 1).

**TABLE 1:** Quantification of the spectral specificity of the hemodynamic DMN.

	**Full**	**Delta**	**Theta**	**Alpha**	**Beta**	**Gamma**
CC	0.26	0.08	0.14	0.64	0.09	0.13
DC	0.13	0.01	0.04	0.58	0.02	0.03

*The spatial correspondence between the fMRI-derived DMN map and the spatial map obtained by correlating the hdEEG-derived DMN time-course with the fMRI signals was assessed using the correlation coefficient (CC) and dice coefficient (DC). The metrics were calculated on the basis of on the group-level maps.*

## Discussion

In this study, we investigated to what extent electrophysiological power modulations can account for hemodynamic activity in the DMN. To this end, we employed an analysis workflow for source-reconstructing hdEEG data acquired during simultaneous fMRI scanning and for studying the relationship between electrophysiological oscillations and hemodynamic activity in the DMN for multiple frequency bands. Our results suggested that alpha power oscillations derived either from EEG sensors data or source-reconstructed EEG network present significant correlation with hemodynamic signals. Concerning the former approach, we found patterns of negative correlations to be significant for voxels belonging to attention-related regions. For the latter analysis, following the detection of hdEEG-derived DMN in multiple frequency bands, the correlation map in alpha band especially displayed a remarkable spatial overlap with DMN regions. This finding supports the hypothesis that DMN spatial configuration is closely associated with the specific neuronal oscillations considered, and that alpha power oscillations might play a dominant role in explaining DMN hemodynamic activity. We will further elaborate on the points above in the following paragraphs.

### Correlation Maps for EEG Sensors Power

Simultaneous EEG–fMRI has already shown to be a viable approach for investigating how changes in electrophysiological oscillations may be linked to hemodynamic functional interactions within and between brain networks (Mantini et al., 2007; Ostwald et al., 2010; Porcaro et al., 2010; Mayhew et al., 2013b, 2017; Lei et al., 2014). Nonetheless, the relationship between electrophysiological oscillations and hemodynamic brain network interactions has been still poorly understood. A large body of simultaneous EEG–fMRI studies was focused on the correlation between EEG sensors alpha power fluctuations and either voxel-by-voxel fMRI signals in the gray matter (Goldman et al., 2002; Laufs et al., 2003a) or fMRI-derived resting state networks (Mantini et al., 2007; Jann et al., 2009; Neuner et al., 2014). The latter, especially, aimed to test whether hemodynamic networks were dependent on global synchronization in specific frequency bands, e.g., DMN mainly by alpha and beta band (Mantini et al., 2007). Several studies reported positive correlations between sensor EEG alpha power fluctuations and fMRI-derived DMN topography (Jann et al., 2009; Knyazev et al., 2011). In these works, it was assumed that positive correlation of electrical oscillatory activity with fMRI signals underlies neuronal synchronization whereas negative correlation underlies neuronal desynchronization. This interpretation is supported by several studies, suggesting that increased alpha activity is associated with increased DMN activity (Jann et al., 2009; Mo et al., 2013), whereas widespread negative correlation with EEG sensors alpha power fluctuations characterize bilateral fronto-parietal network encompassing brain regions involved in attention processes (Laufs et al., 2003a; Moosmann et al., 2003; Haufe et al., 2018). The prevalence of alpha oscillations in the EEG has been unambiguously related to vigilance, but its correlates with BOLD signal still showed diverging results. In particular, other studies reported that the alpha power did not increase in the regions belonging to the DMN (Chang et al., 2013), but increased within attention-related brain regions (Sadaghiani et al., 2012). Also, regions of the cingulo-opercular (Power et al., 2011) or salience (Seeley et al., 2007) network, involved in sustained maintenance of non-selective alertness, were identified. This suggests that EEG alpha power fluctuations emerge when subjects are less focused on performing a goal directed task, as it occurs during resting state, but does not give a definitive answer concerning the relationship between EEG power and fMRI signals. Our findings are consistent with previous EEG–fMRI studies that reported widespread negative occipital alpha power correlation with voxel-by-voxel fMRI signals in brain regions belonging to attention-related networks (Laufs et al., 2003a, b; Gonçalves et al., 2006; de Munck et al., 2007). Interestingly, these studies showed that the correlation patterns did not largely depend on the EEG sensors chosen for the EEG–fMRI analysis (Laufs et al., 2003b; Gonçalves et al., 2006; de Munck et al., 2007). Also, we reported a pronounced positive correlation in the MPFC, a region notably involved in mediating the processing of information from internal or external sources, which was previously described for occipital alpha power analysis (Scheeringa et al., 2012).

### Correlation Maps for hdEEG Source Reconstructed DMN

In this study, we contributed to elucidate the electrophysiological basis of hemodynamic activity in brain networks. In particular, we substantiated the feasibility of hdEEG for detecting resting state networks, by further improving the hdEEG processing pipeline (Liu et al., 2017) for enabling reliable resting state networks identification also from data simultaneously acquired with fMRI.

By correlating voxel-by-voxel fMRI signals with the time-course associated with the hdEEG-derived DMN spatial map, we found a positive correlation pattern displaying a spatial distribution depicting DMN areas in alpha and full band (Figure 4 and Table 1). Following a similar approach, a previous study, exclusively considering full band power correlation, reported significant correlations especially in MPFC (Yuan et al., 2016), and only partially to other DMN nodes. In our study, we also extended this approach to all frequency bands to investigate how different brain rhythms contribute to DMN functioning. Notably, the temporal profiles of DMN activity in alpha and full band were the only ones displaying spatial pattern depicting brain regions typically associated with DMN (see Figure 4). Taken together, our findings suggest that DMN spatial configuration closely relates to the specific neuronal oscillations considered and that alpha rhythm especially contribute to DMN activity (Samogin et al., 2019). By showing that the spatial pattern derived by alpha power correlation with the fMRI signals yields large overlap with DMN regions (see Table 1), this study further corroborates the idea that alpha-band oscillations at rest support DMN functioning (Mantini et al., 2007; Samogin et al., 2019).

## Conclusion

By using resting-state data from simultaneous EEG–fMRI, we investigated the neural correlates of hemodynamic activity in the DMN for multiple frequency bands, and we provided new insights into DMN functioning. In particular, we found that the DMN spatial configuration depends on the specific neuronal oscillations considered, and that alpha rhythm is mainly associated with DMN activity, especially when source-reconstructed data are analyzed. Importantly, our approach for hdEEG–fMRI data integration can be applied to investigate the neurophysiological basis of human brain networks, and open new ways for defining a coupling model between EEG and fMRI data. The spatial arrangement of network correlation maps might be used as a starting point for identifying functionally relevant cortical sites in the network of interest. This might be used to disentangle the relationship between source-reconstructed electrophysiological activity and hemodynamic measurements, and distinguish the frequency bands that support hemodynamic functional interactions between distant brain areas.

## Data Availability Statement

The datasets generated for this study are available on reasonable request to the corresponding author.

## Ethics Statement

This study was carried out in accordance with the recommendations of the Medical Ethics Committee of the UZ Leuven with written informed consent from all subjects. All subjects gave written informed consent in accordance with the Declaration of Helsinki. The protocol was approved by the Medical Ethics Committee of the UZ Leuven.

## Author Contributions

MM and DM designed the research and developed the method. MM analyzed the data with the support of GA and CP and wrote the first draft of the manuscript, which all authors revised and approved. DM directed the study. All authors participated to the scientific discussion.

## Conflict of Interest

The authors declare that the research was conducted in the absence of any commercial or financial relationships that could be construed as a potential conflict of interest.

## Footnotes

1 http://www.fil.ion.ucl.ac.uk/spm/software/spm12/

2 https://sccn.ucsd.edu/eeglab/

3 https://www.mrt.uni-jena.de/simbio
